# Melatonin-Mediated Development of Ovine Cumulus Cells, Perhaps by Regulation of DNA Methylation

**DOI:** 10.3390/molecules23020494

**Published:** 2018-02-23

**Authors:** Yi Fang, Shoulong Deng, Jinlong Zhang, Haijun Liu, Yihai Li, Xiaosheng Zhang, Yixun Liu

**Affiliations:** 1 Animal Husbandry and Veterinary Research Institute of Tianjin, Tianjin 300381, China; 17743112414@163.com or fangyi@iga.ac.cn (Y.F.); jlzhang1010@163.com (J.Z.); liuhj67@126.com (H.L.); tjliyihai@foxmail.com (Y.L.); 2 Jilin Provincial Key Laboratory of Grassland Farming, Northeast Institute of Geography and Agoecology, Chinese Academy of Sciences, Changchun, Jilin 130062, China; 3 State Key Laboratory of Stem Cell and Reproduction Biology, Institute of Zoology, Chinese Academy of Science, Beijing 100101, China; dengsl@ioz.ac.cn

**Keywords:** melatonin, cumulus cells, methylation, methyltransferases, lamb

## Abstract

Cumulus cells of pre-pubertal domestic animals are dysfunctional, perhaps due to age-specific epigenetic events. This study was designed to determine effects of melatonin treatment of donors on methylation modification of pre-pubertal cumulus cells. Cumulus cells from germinal vesicle stage cumulus oocyte complexes (COCs) were collected from eighteen lambs which were randomly divided into control group (C) and melatonin group given an 18 mg melatonin implant subcutaneous (M). Compared to the C group, the M group had higher concentrations of melatonin in plasma and follicular fluid (*p* < 0.05), greater superovulation, a higher proportion of fully expanded COCs, and a lower proportion of apoptotic cumulus cells (*p* < 0.05). Real-time PCR results showed that melatonin up-regulated expression of genes *MT1*, *Bcl2*, *DNMT1*, *DNMT3a* and *DNMT3b*, but down-regulated expression of genes *p53*, *Caspase 3* and *Bax* (*p* < 0.05). Furthermore, melatonin increased FI of FITC (global methylation level) on cumulus cells (*p* < 0.05). To understand the regulation mechanism, the *DNMTs* promoter methylation sequence were analyzed. Compared to the C group, although there was less methylation at two CpG sites of *DNMT1* (*p* < 0.05) and higher methylation at two CpG sites of *DNMT3a* (*p* < 0.05), there were no significant differences in methylation of the detected *DNMT1* and *DNMT3a* promoter regions. However, there were lower methylation levels at five CpG sites of *DNMT3b*, which decreased methylation of detected *DNMT3b* promoter region on M group (*p* < 0.05). In conclusion, alterations of methylation regulated by melatonin may mediate development of cumulus cells in lambs.

## 1. Introduction

Evaluation of the reproductive potential of pre-pubertal animal can solve the lack of embryonic origin for the commercialization of embryo transfer, significantly reduce the cost of embryo production and greatly improve the breeding and production efficiency [1]. However, developmental capacity of oocytes from pre-pubertal animals is reduced compared to their adult counterparts. Besides incomplete or deficient maturation of cytoplasm [2], altered protein synthesis [3], and impaired metabolism [4], cumulus cell dysfunction also contribute. 

Cumulus cells are considered to have important roles in oocyte maturation by: (1) keeping the oocyte under meiotic arrest; (2) participating in the induction of meiotic resumption; (3) supporting cytoplasmic maturation. These key cumulus cell functions during oocyte maturation are attributable to their elaborate gap junctional network and to their specific metabolic capacities [5,6]. Pre-pubertal cumulus cells have a prominent nucleus and limited cytoplasm with high transcriptional activity, communicating with each other by a few short processes, with limited processes reaching the oocyte [7,8]. Young donors had disturbed DNA methylation processes due to insufficient methyltransferases during oocyte maturation and embryo development [9,10,11], although methylation characteristics of the cumulus cells remain unclear. It was, therefore, hypothesized that pre-pubertal cumulus cells were epigenetically immature because DNA methylation modifications lacked adult-like cumulus cell methylation patterns.

In animals that exhibit seasonal reproduction, such as sheep, photoperiodic information is conveyed to the reproductive neuroendocrine system by circadian secretion of melatonin from the pineal gland [12]. A night-time increase in plasma melatonin concentrations has been reported to occur within 1–6 week-old mammals after birth [13]. In addition to being an antioxidant, melatonin is likely an epigenetic regulator, as it and its metabolites have similar structures and hypothetically could regulate DNA methyltransferases (DNMTs), either by masking target sequences or by blocking the active site of the enzyme [14,15]. Melatonin is a highly lipophilic and somewhat hydrophilic molecule that easily crosses cell membranes, reaching intracellular organelles including the nucleus [16] to accumulate in the nucleus and it interacts with specific nuclear binding sites [17]. So-called nuclear receptors for melatonin have been identified and some studies have linked them to melatonin control of cell growth and differentiation [18]. Furthermore, melatonin binding sites were identified not only in granulosa cells from preovulatory follicles [19,20], but also in cumulus and granulosa cells [21]. Two distinct receptor subtypes *MT1* and *MT2* genes have been cloned and mapped in several animal species [22,23,24]. In mammals, MT1 seemed to be involved more in regulation of reproductive activity than MT2 [25]. Although, addition of melatonin during in vitro maturation (IVM) protected cumulus cells from DNA damage [26], little information is available about effects of melatonin on pre-pubertal cumulus cells in vivo, especially with regards to epigenetic modification. 

The objective was to investigate potential epigenetic mechanisms improving cumulus cells quality in prepubertal lambs by determining whether melatonin treatment altered gene expression of key enzymes and methylation modification. 

## 2. Results

### 2.1. Effects of Exogenous Melatonin on Plasma and Follicular Fluid Melatonin Concentrations

**Figure 1:**
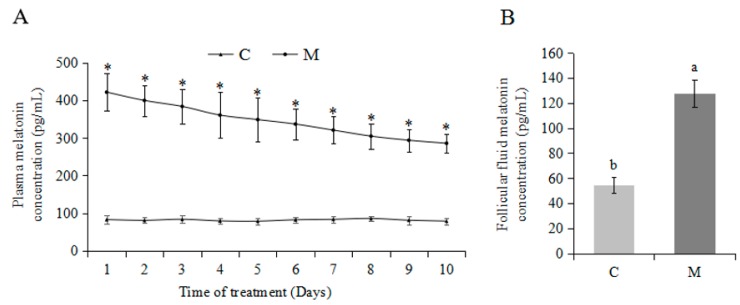
Melatonin concentrations in plasma and follicular fluid. (*n* = 9). * *p* < 0.05 in Figure 1A; a,b in Figure 1B, for columns, means without a common superscript differed (*p* < 0.05). (**A**) Melatonin concentrations of plasma, (**B**) melatonin concentrations of follicular fluid.

Compared to the C group, the M group had higher concentrations (*p* < 0.05) of melatonin in both plasma and follicular fluid (Figure 1).

### 2.2. Effects of Melatonin on Superovulaton and Cumulus Cells Expansion

**Figure 2:**
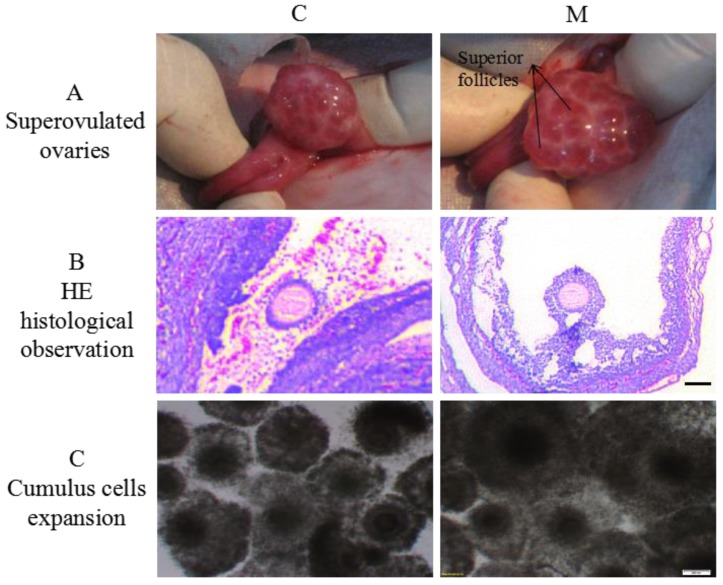
(**A**) Ovaries after superovulation, (**B**) HE histological appearance (bar = 500 μm) and (**C**) expansion of cumulus cell after IVM (×10).

Compared to the C group, the M group had a better superovulatory response (*p* < 0.05; Table 1; Figure 2A,B), lower proportions of not expanded and partially expanded cumulus oocyte complexes (COCs), and higher proportion of fully expanded COCs (*p* < 0.05; Table 2; Figure 2C).

### 2.3. Effects of Melatonin on Apoptosis and Expression of Related Genes in Cumulus Cells 

**Figure 3:**
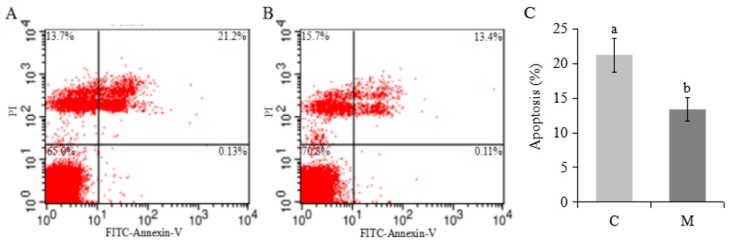
Effects of melatonin on cumulus cells apoptosis. Flow cytometry (**A**: C group; **B**: M group); mean ± SEM cumulus cells apoptosis (**C**). The experiment was repeated three times; Data presented as mean ± SEM; a,b in Figure 3C, for columns, means without a common superscript differed (*p* < 0.05).

**Figure 4:**
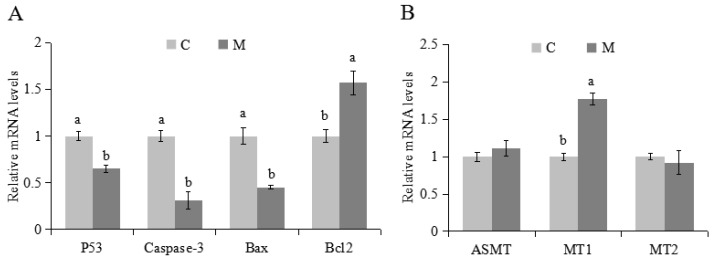
Effects of melatonin on expression of related genes in cumulus cells. Expression of related apoptosis genes in cumulus cells (**A**); expression of melatonin synthetase and receptors genes (**B**). The experiment was repeated three times; data presented as mean ± SEM; a,b for adjacent columns, means without a common superscript differed (*p* < 0.05).

There was a lower proportion of apoptotic cumulus cells in the M versus C groups (*p* < 0.05; Figure 3A–C). Melatonin increased mRNA expression of *Bcl2* and *MT1*, but decreased *P53*, *Caspase 3* and *Bax* (*p* < 0.05; Figure 4A,B). Melatonin had no effect on *ASMT* or *MT2* (Figure 4B). 

### 2.4. Methylation Modifications in Cumulus Cells 

**Figure 5:**
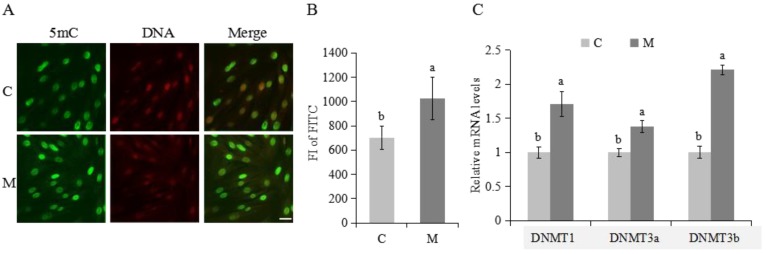
Abundance of 5-methylcytosine (5mC) and gene expression of *DNMTs* in cumulus cells collected from C and M groups; (**A**) Representative immunofluorescence images of 5mC (green), and propidium iodide-stained nuclei (red). Scale bars, 20 μm. (**B**) global 5mC DNA in cumulus cells. (**C**) relative mRNA levels of *DNMTs*. The experiments were repeated three times; data presented as mean ± SEM; a,b in Figure 5B,C, for adjacent columns, means without a common superscript differed (*p* < 0.05).

Melatonin increased FI of FITC (methylation marker) and increased mRNA expression of *DNMT1*, *DNMT3a* and *DNMT3b* on cumulus cells (*p* < 0.05; Figure 5A–C). 

**Figure 6:**
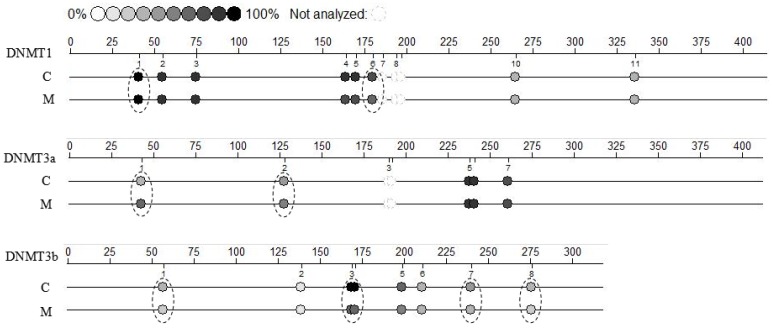
Methylation sequencing in cumulus cells; the experiment was repeated three times; loci within a circle differed (*p* < 0.05).

Compared to the C group, there were no significant differences in methylation of *DNMT1* and *DNMT3a* promoter CpG region, and decreased methylation of *DNMT3b* promoter CpG region on melatonin group (*p* < 0.05; Appendix: Table A2, Table A3 and Table A4). There were lower methylation levels at two CpG sites (CpG40, CpG179) of *DNMT1* (*p* < 0.05; Figure 6, Appendix: Table A2) and at five CpG sites (CpG56, CpG168:170, CpG239, CpG275) of *DNMT3b* (*p* < 0.05; Figure 6, Appendix: Table A4), and higher methylation levels at two CpG sites (CpG42, CpG127) of *DNMT3a* (*p* < 0.05; Figure 6, Appendix: Table A3) in the M group.

## 3. Discussion

In addition to its antioxidant properties, melatonin regulates gene expression [15] and therefore has epigenetic effects. In the present study in prepubertal lambs, melatonin promoted cumulus cells development, and regulated related genes expression, perhaps due to alterations of methylation modification.

In 1-mo-old superovulated lambs, exogenous melatonin significantly increased melatonin concentrations in both plasma and follicular fluid, enhanced superovulation and cumulus cells expansion. Melatonin has improved the quality of human [27], porcine [28,29], bovine [24,26,30,31], and murine [32,33] oocytes (with or without cumulus cells), embryos, and cumulus cells by reducing cytoplasmic ROS concentrations or DNA damage. Melatonin concentrations in the follicular fluid vary with follicle size, increasing with an increased follicular diameter in humans [34]. High melatonin concentrations in follicular fluid help to prevent atresia, enabling full development of preovulatory follicles and protecting adjacent cells and the oocyte from free radicals [35,36]. Perhaps melatonin modulates the follicular response to LH by elevating mRNA expression of LH receptors in granulosa and cumulus cells [37]. Additionally, melatonin promotes follicular development by increasing production of insulin-like growth factor I which stimulates mitogenic growth of cumulus cells [38]. Although previous in vivo studies demonstrated exogenous FSH may mask effects of melatonin on follicular development, exogenous melatonin tended to increase the number of developing follicles [39]. Perhaps the role of melatonin in regulation of germ cell development is species- or age-specific.

Mammalian cumulus cells have very important roles during oocyte growth and maturation. They supply nutrients [40] and/or messenger molecules for oocyte development [41,42], and to mediate effects of hormones on oocytes [43]. Moreover, cumulus cells expansion is considered an important marker for oocyte maturation and is essential for fertilization, subsequent cleavage, and blastocyst development [44]. Our results confirmed that melatonin had a potentially significant effect on the degree of lamb cumulus cells expansion. The same promoting effects of melatonin on cumulus cell expansion were reported in bovine [24] and porcine oocytes [45]. 

It is well established that melatonin induces apoptosis in cancer-like cells [46,47], whereas in normal cells it prevents apoptosis [48]. A lower proportion of apoptotic cumulus cells in this study indicated that melatonin had a beneficial effect, confirmed by decreasing expression of genes *P53*, *Caspase 3* and *Bax*, and increasing expression of *Bcl2* genes. In general, it is believed that the balance between Bax and Bcl-2 determines the propensity of cells to respond to a given insult by apoptosis or survival [49]. Melatonin shifts the balance towards a protective state by suppressing pro-apoptotic *Bax* and inducing expression of *Bcl-2* [50,51].

*MT1* and *MT2*, expressed in follicular cumulus, mural granulosa cells and oocytes [52], contribute to regulation of follicular development, proliferation, and influence hormone signaling [53]. In the present study, up-regulation of *MT1* gene expression in lambs indicated that the positive effect of melatonin on cumulus cells may be mediated by MT1. Therefore, we speculated that some effects of melatonin on lamb cumulus cells were likely to be directly mediated by receptor mechanisms. Moreover, the unchanged expression of *MT2* in cumulus cells supported previous reports suggesting that MT1 was more important than MT2 for regulation of reproduction [25]. In the absence of alterations of gene expression of *ASMT*, we inferred that there was production of melatonin secretion as early as 1 month of age in lambs and consequently, exogenous melatonin treatment may not affect endogenous melatonin secretion on cumulus cells.

Pre-pubertal germ cells are epigenetically immature, and epigenetics has been proposed to be involved in acquisition of full developmental or proliferative competence. Methylation sequence changes are associated with donor age [10], with less DNA methylation in pre-pubertal germ cells [6,11]. The positive effect of melatonin on cumulus cells in this study may be related to increasing global methylation. There is direct evidence of epigenetic actions for melatonin, including significant increases in mRNA expression for various HDAC isoforms and elevated histone H3 acetylation in neural stem cell lines [54]. Methylation actions of melatonin suggest epigenetic regulation at a co-regulator level rather than selective enzymatic inhibition or activation [54]. Several studies indicated that melatonin inhibited *COX-2* and *iNOS* transcriptional activation, suggested to be an epigenetic action [55]. This had significant benefits in treatment of experimental hyperglycemia mediated by epigenetic regulation [56]. Furthermore, this also involved anti-inflammatory actions, controlled by activation of the NF-κB and AP-1 family, which induces various epigenetic processes by altering chromatin structure [57,58].

The higher level of global methylation in this study may due to overexpression of *DNMT1*, *DNMT3a* and *DNMT3b* genes. Despite uncertainty regarding how melatonin can regulate a variety of genes such as *DNMTs*, *MT1*, apoptosis genes but always in the right direction (some inhibition, some activation), these actions may be due to an epigenetic mechanism [59,60]. Most genes are either active or inactive under steady-state conditions and in order to change their status (e.g., from on to off and vice versa), several epigenetic modifications such as histone modifications and DNA methylations are required. Accumulating data in the turning on/off of genes and gene regulation by melatonin have converged with discoveries of epigenetic mechanisms. Although there were lower methylation level at two CpG sites (CpG40, CpG179) of *DNMT1* and higher methylation level at two CpG sites (CpG42, CpG127) of *DNMT3a* after melatonin treatment, there were no differences in methylation of detected promoter region of *DNMT1* and *DNMT3a*. Melatonin decreased methylation of the *DNMT3b* promoter CpG region, reflected at five CpG sites (CpG56, CpG168:170, CpG239, CpG275) after melatonin treatment. Melatonin regulated *DNMTs* gene induction by methylation and also seemed to recruit basal transcriptional machinery to the promoter region of DNMT-related genes. In conclusion, some of the positive effects of melatonin on cumulus cells was attributed to regulation of methylation events.

## 4. Materials and Methods

All procedures involving animals were approved by the Chinese Academy of Science Animal Care and Use Committee (Grant No. 20120208). All chemicals were purchased from Sigma Aldrich (St. Louis, MO, USA), unless otherwise indicated.

### 4.1. Animals

Eighteen 4-wk-old Hu-sheep lambs were obtained from the Institute of Animal Husbandry and Veterinary Medicine (Tianjin, China). Lambs were housed in a 12L:12D light-dark cycle, in a temperature-controlled (25 °C) room, with feed and water available *ad libitum*. Lambs were randomly and equally allocated into two groups, the control group (C) and the melatonin implantation group (M). Lambs in the M group had an implant containing 18 mg melatonin (Melovine^®^, CEVA Salud Animal, Barcelona, Spain), placed subcutaneous at the base of the left ear. 

### 4.2. Donor Superovulation

**Figure A1:**
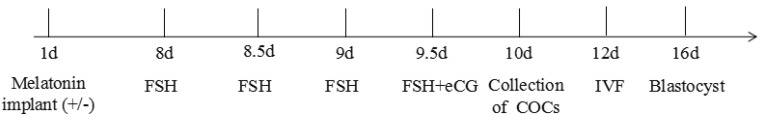
Superovulation scheme (*n* = 9).

All lambs were given 125 IU of follicle-stimulating hormone (FSH, Sansheng, Ningbo, China), injected im on two occasions, 24 h apart. In the M group, the first dose of FSH was given 7 days after placement of the melatonin implant. In addition, 250 IU of equine chorionic gonadotrophin (eCG, Sansheng, Ningbo, China) was given im at the time of the last FSH treatment (Appendix, Figure A1. Superovulation scheme). 

### 4.3. Blood Sampling and Hormone Assay

Every day during the superovulation period, blood samples (3 mL) were collected for ten consecutive days (Appendix, Figure A1. Superovulation scheme) by jugular venipuncture using heparinized vacutainers (Beyotime, Jiangsu, China) to detect plasma melatonin concentration. To minimize effects of circadian rhythms, sample collection was consistently done at night (2:0) in resting conditions, always by the same operator. Blood was placed into chilled tubes (Beyotime) containing 0.125 M EDTA and 0.025 M *o*-phenanthroline, centrifuged (1000× *g* at 4 °C for 15 min), and plasma immediately extracted and stored at −20 °C. A commercial enzyme-linked immunosorbent assay for melatonin (EIA 1431, sensitivity 0.977 mIU/mL; DRG Diagnostics, Marburg, Germany), was done according to manufacturer's instructions. Intra- and inter-assay coefficients of variations were 4.0 and 13.7%, respectively. 

### 4.4. The Cumulus Oocyte Complexes Collection

At 14 h after eCG, COCs were collected by aspiration. Briefly, lambs were anaesthetized with acepromazine maleate (0.05 mg/kg body weight) and sodium pentothal (10 mg/kg body weight), the abdomen opened on the left or right lower 5–7 cm of the breast with 3–4 cm incision. Ovaries were exposed and COCs were aspirated from visible follicles (2–5 mm in diameter) using a 10-mL syringe equipped with an 18 gauge needle and containing HEPES-buffered DMEM/F-12. Following aspiration, the ovaries and tips of the uterine horns were washed extensively with saline to minimize adhesions. The aspirated fluid was examined under a phase-contrast microscope (Olympus BX60, Olympus, Japan) and only COCs with homogeneous granular cytoplasm and at least three or four layers of compact cumulus cells were selected. The COCs from nine sheep of each group were collected together, then were randomly selected for following experiments (Appendix, Table A1). Melatonin concentrations in follicular fluids were determined by ELISA (same assay used for plasma).

### 4.5. Histology Staining

After COCs being collected, eight ovaries were removed from four donors in each group. Samples were placed overnight in 4% buffered formaldehyde (37% formaldehyde, Merck, Darmstadt, Germany). Fixed tissues were embedded in paraffin blocks and the whole ovary was sectioned serially at 4 μm thickness. Three sequential sections were put on each slide. Every second or third slide was stained with hematoxylin and eosin.

### 4.6. In Vitro Maturation (IVM)

The maturation medium contained 20% (*v*/*v*) heat-inactivated estrous sheep serum, 10 μg/mL FSH, 10 μg/mL luteinizing hormone, 10 ng/mL epidermal growth factor, and 1 μg/mL estradiol-17β in TCM199 medium. Each drop contained 50 μL in vitro maturation medium that was equilibrated in a CO_2_ incubator (Thermo, Waltham, MA, USA) for 2 h before the COCs were placed in the medium. Each group of 15 oocytes was cultured in a 50 μL drop of maturation medium in humidified air with 5% CO_2_ at 39 °C for 24 h. The degree of cumulus cells expansion was subjectively assessed under a phase-contrast microscope (Olympus BX60, Tokyo, Japan) after 22 h of IVM; COCs were classified as not expanded, partially expanded (outer layer of cells was loosened), or fully expanded (all cumulus cells were loosened), as described [61].

### 4.7. In Vitro Fertilization (IVF) and In Vitro Culture

The COCs were incubated with 0.1% hyaluronidase to dissociate cumulus cells after IVM, then the oocytes were fertilized with the same ram fresh sperm in synthetic oviduct fluid medium containing 20% (*v*/*v*) estrous sheep serum and 10 μg/mL heparin (in IVF medium) in the incubator. The IVF drops were prepared and equilibrated in an incubator for 2 h before insemination. The volume of each drop was 40 μL. Groups of up to five oocytes were transferred into the IVF drops. Sperm concentration was calculated using a hemocytometer (SDM1, Minitube, Diefenbach, Germany) and diluted to 1 × 10^6^ cells/mL with IVF medium. Subsequently, there was 10 μL of the sperm suspension added to the 40 μL IVF drops. The gametes were co-incubated at 39 °C in a 5% CO_2_ humidified air atmosphere for 22 h. 

At approximately 22 h following the addition of semen to the culture medium, presumptive zygotes were washed in synthetic oviductal fluid (SOF) medium to remove sperm before being transferred to 50 μL culture droplets of SOF supplemented with 1% (*v*/*v*) Basal Medium Eagle-essential amino acids, 1% (*v*/*v*) Modified Eagle Medium-nonessential amino acids, 1 mM glutamine, and 6 mg/mL fatty acid-free BSA under mineral oil. The contents of the dishes were incubated at 39 °C in a 5% CO_2_, 5% O_2_, 90% N_2_ humidified atmosphere. Cleavage and hatching rates were recorded at 48 h and 7 d post-IVF, respectively. 

### 4.8. Cumulus Cell Apoptosis 

A portion of COCs were washed two or three times in HEPES-buffered DMEM/F-12, cumulus cells dissociated by incubation with 0.1% hyaluronidase for 5 min and continuous pipetting to isolate cumulus cells from oocytes. Thereafter, the cumulus cells suspension was centrifuged at 1000× *g* for 5 min and the supernatant decanted. This was repeated two or three times, using phosphate-buffered saline (PBS) to wash the cellular pellet.

Apoptosis was assessed using a commercial kit (FITC Annexin V Apoptosis Detection Kit I; Becton Dickinson, Sunnyvale, CA, USA), in accordance with manufacturer’s instructions. Cells were washed with PBS and centrifuged three times at 800× *g* for 5 min. Then, 100 µL of PBS was added into each centrifuge tube, followed by addition of 5 µL of FITC solution and 5 μL of propidium iodide (20 μg/mL). Cells were incubated at room temperature in the dark for 15 min. Samples were assayed within 2 h using a flow cytometer (Becton Dickinson). Data for 20,000 cells per sample were stored in the list mode using FACS Analyzer flow cytometry software 3.0 (Becton Dickinson); thereafter, these data were passed through a Hewlett Packard Consort 30 system (Palo Alto, CA, USA) and analyzed by SuperCyt Analyst 3 software (Sierra Cytometry, Reno, NV, USA). Apoptotic and dead cells were distinguished by staining with propidium iodide and FITC. All experiments were performed in biological triplicates, and data were representative of at least three independent experiments.

### 4.9. Global Methylation Analysis

After cumulus cells were isolated, the suspension was placed in a cell culture plate, DMEM/F-12 (penicillin & streptomycin (Hyclone, Logan, Utah, USA) and 10% fetal calf serum (BI, Tel Aviv, Israel)) was added to the plate, and the plate incubated at 38.5 °C with 5% CO_2_ in humidified air. Cumulus cells used for immunocytochemical staining were permeabilized with 1% Triton X-100 (Beyotime) in phosphate-buffered saline (PBS) for 30 min and then treated in 2 M HCl (Beyotime) for 30 min at 25 °C. Non-specific binding was inhibited with 0.1% BSA (Beyotime) for 30 min at room temperature, and then cells were incubated with anti-5meC antibodies (1:500; Epigentek Group Inc., Farmingdale, NY, USA) at 4 °C overnight. Cumulus cells were extensively washed and then probed with fluorescein isothiocyanate-conjugated anti-mouse IgG (1:100; Santa Cruz Biotechnology, Inc., Dallas, TX, USA) for 1.5 h at 37 °C. The DNA was visualized by counterstaining with 10 μg/mL propidium iodide for 10 min. After extensive washing, cumulus cells were incubated in PBS containing 10% triethylenediamine. Fluorescence was detected with an Olympus BX40 spectral confocal scanning microscope at excitation wavelengths of 488 and 543 nm. System settings were held constant for all examinations. Fluorescence intensity was quantified using FV10-ASW 3.0 Free Viewer software (Olympus). The mean fluorescence pixel value was measured from at least 100 cells per plate and five plates per sample.

### 4.10. RNA Purification and qRT-PCR

Total RNA was extracted using TRIzol reagent (Invitrogen, Carlsbad, CA, USA), and RNase-free DNase was used to remove genomic DNA. Integrity and concentration of RNA were determined by measuring absorbance at 260 nm. Total RNA (1.0 µg) from each sample was re-suspended in a 20 µL final volume of reaction buffer, containing 25 mM Tris-HCl, 37.5 mM KCl, 10 mM dithiothreitol, 1.5 mM MgCl_2_, 10 mM of each dNTP, and 0.5 mg oligo (dT)_15_ primers to synthesize the cDNA. After the reaction mixture reached 42 °C, 20 units of reverse transcriptase was added to each tube, and the sample incubated for 1 h at 42 °C. Reverse transcription was stopped by denaturing the enzyme at 95 °C. The final PCR mixture contained 2.5 µL cDNA, 1× PCR buffer, 1.5 mM MgCl_2_, 200 µM dNTP mixture, 1 U of Taq DNA polymerase, 1 µM sense and antisense primers, and 5.0 µL sterile water. The qRT-PCR was conducted using the CFX96TM Real-Time PCR Detection System (Bio-Rad, Hercules, CA, USA) under standard conditions. Transcripts were quantified in triplicate for each sample, and β-actin was used as a reference. Expression levels were calculated using the comparative Ct (2^−ddCt^) method [62]. Primers used are listed in Table 3.

### 4.11. DNA Methylation Sequence

**Figure 7:**
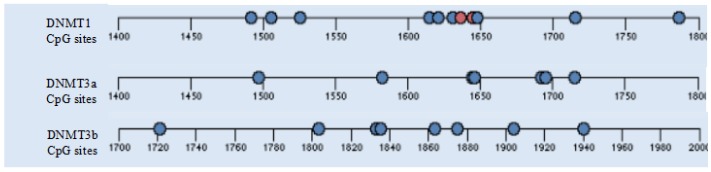
Gene structures of detected promoter region of *DNMT1*, *DNMT3a* and *DNMT3b*. Locations of CpG sites in this study indicated in blue were analyzed, whereas those indicated in red were either not uniquely discriminated in the spectra or had low call rates.

An aliquot (1 µm) of DNA was treated with bisulfite using an EpiTect Bisulfite Kit (Qiagen, Hilden, Germany). Then, DNA methylation of CpG sites (Figure 7) in the promoter region of the DNMT1, DNMT3a, DNMT3b gene were analyzed using EpiTYPER (MassARRAY system; Agena Biosciences, Santiago, CA, USA) according to the manufacturer’s instructions. DNMT1 forward (aggaagagagTGTAAGGTAAGGGTTTAATTTTATTTTT) and reverse (cagtaatacgactcact atagggagaaggctCCAACCTCAATTTCCTCATCTATAA) primers corresponding to product 394 size, coverage 9/11 CpG, DNMT3a forward (aggaagagagTTAGAGGGTGTTTTGGAAAGGGTAA) and reverse (cagtaatacgactcactatagggagaaggctAACAAAAACAAATATTTCCTATATACACC) primers corresponding to product 392 size, coverage 7/7 CpG, DNMT3b forward (aggaagagagGTTGTTATGGAGAGGAGAGAAGTTG) and reverse (cagtaatacgactcactatagggaga aggctACCAACACCCAAAACAAAAAAA) primers corresponding to product 298 size, coverage 8/8 CpG, were designed using EpiDesigner (Agena Bioscience), and spectrum characteristics were validated with RSeqMeth (Figure 7). Cycling conditions were: denaturation (94 °C for 4 min) then 45 cycles of amplification (94 °C for 20 s, 56 °C for 30 s, and 72 °C for 1 min) and a final extension step at 72 °C for 3 min. Samples were electrophoresed using 2% (*w*/*v*) agarose gel to confirm amplification. The CpG sites were unambiguously interrogated, and their genomic locations detailed (Figure 7). Mass spectra methylation ratios were generated using EpiTYPER v1.2 (Agena Biosciences). Finally, reliability of the methylation assay was confirmed using Epitect unmethylated (0%) and methylated (100%) DNA samples (Qiagen) as positive controls. For each participant, average DNA methylation values were calculated by averaging across a total of CpG cites. All experiments were performed in biological triplicates, and data were representative of three independent experiments.

### 4.12. Statistical Analyses

All data (except those in Table 2 and maturation rate, cleavage rate, blastocyst rate in Table 1) were presented as mean ± SEM and analyzed with one-way ANOVA, followed by Duncan’s test, using SPSS 18.0 statistical software (SPSS Inc., Chicago, IL, USA). Values in Table 2 and maturation rate, cleavage rate, blastocyst rate in Table 1 were analyzed using a Chi-square test. For all analyses, *p* < 0.05 was considered significant. Data are expressed as mean ± SEM.

## Figures and Tables

**Table 1:** Effect of melatonin on lamb superovulation.

Groups	Mean ± SEM No. Follicles ≥ 2 mm (*n* = 9)	Mean ± SEM No. Oocytes (*n* = 9)	IVF		
			Maturation Rate (%) (No. Mature/No. Total)	Cleavage Rate (%) (No. Cleavage/No. Mature)	Blastocyst Rate (%) (No. Blastocyst/No. Cleavage)
C	79.4 ± 9.22 ^b^	55.2 ± 4.29 ^b^	71.8 (155/216) ^b^	52.3(81/155) ^b^	7.41(6/81) ^b^
M	106 ± 14.9 ^a^	86.3 ± 7.77 ^a^	79.5 (291/366) ^a^	68.4(199/291) ^a^	14.1(28/199) ^a^

^a,b^ Within a column, means without a common superscript differed (*p* < 0.05).

**Table 2:** Effect of melatonin on cumulus cells expansion.

Groups	No. COCs Examined	No. (%) Oocytes with Cumulus Cells		
		Not Expanded	Partially Expanded	Fully Expanded
C	216	39 (18.0) ^a^	67 (31.0) ^a^	110 (51.0) ^b^
M	366	29 (8.00) ^b^	59 (16.1) ^b^	278 (76.0) ^a^

^a,b^ Within a column, means without a common superscript differed (*p* < 0.05).

**Table 3:** Primer sequences and conditions.

Gene	Primer Sequence	T annealing (°C) × No. Cycles	Fragment Size (bp)
*β-Actin*	5′GTCATCACCATCGGCAATGA 3′CGTGAATGCCGCAGGATT	60 × 35	159
*ASMT*	5′TCATTTTCCTGAGTGCGTTG 3′CTCCCAGGTTCTCTTTGCTG	58 × 35	205
*MT1*	5′GGAGGGTGAAACCTGACGAC 3′CCCAGCAAATGGCAAAGAGG	57 × 35	99
*MT2*	5′GGCTCCGTCTTCAACATCACC 3′GCAGAAGGACCAGCAGGGTG	60 × 35	145
*P53*	5′GAAGACCTACCCTGGCAATTAC 3′AGAACAGCTTGTTAAGGGAAGG	60 × 35	103
*Bax*	5′GGCTGGACATTGGACTTCCTTC 3′TGGTCACTGTCTGCCATGTGG	57 × 35	112
*Caspase 3*	5′CAGACAGTGGTGCTGAGGATGA 3′GCTACCTTTCGGTTAACCCGA	56 × 35	211
*Bcl2*	5′GACTGACACTGAGTTTGGCTACG 3′GAGTCCTTTCCACTTCGTCCTG	58 × 35	152
*DNMT1*	5′CGCATGGGCTACCAGTGCACCTT 3′GGGCTCCCCGTTGTATGAAATCT	58 × 35	158
*DNMT3a*	5′CAACGGAGAAGCCTAAGGTCAA 3′TTGAGGCTCCCACAAGAGATG	60 × 35	244
*DNMT3b*	5′GACTCATTGGAGGACCAGCTGAAGC 3′CAGCACCTCCAGGCACTCCACACAG	59 × 35	130

## Appendix

**Table A1:** Number of COCs used per group for different tests (*n* = 9).

Groups	Sum No. COCs	No. COCs Examined			
		Expansion	Apoptosis	RT-PCR	Methylation
C	497	216	69	81	131
M	777	366	92	88	231

**Table A2:** Mean ± SEM effect of melatonin on methylation sequence of *DNMT1*.

Group	CpG Position (%)										Sum (%)
	40	54	74	163:169	179	185	193	196	264	335	
C	100 ± 5.55 ^a^	84.2 ± 9.13	88.5 ± 8.02	77.1 ± 5.73	80.2 ± 9.09 ^a^	NA	NA	NA	35.4 ± 4.24	35.1 ± 3.51	70.6 ± 8.71
M	79.7 ± 9.01 ^b^	96.6 ± 11.6	81.9 ± 6.33	80.6 ± 9.11	62.4 ± 5.83 ^b^	NA	NA	NA	35.9 ± 2.89	35.6 ± 3.77	67.6 ± 7.49

The experiment was repeated three times; data presented as mean ± SEM; ^a,b^ within a column, means without a common superscript differed (*p* < 0.05).

**Table A3:** Mean ± SEM effect of melatonin on methylation sequence of *DNMT3a*.

Group	CpG Position (%)					Sum (%)
	42	127	189:191	237:240	260	
C	47.5 ± 4.02 ^b^	44.3 ± 5.05 ^b^	NA	85.4 ± 8.08	77.4 ± 6.48	63.3 ± 7.34
M	64.1 ± 7.17 ^a^	53.2 ± 4.13 ^a^	NA	81.9 ± 6.91	79.7 ± 7.05	69.3 ± 5.77

The experiment was repeated three times; data presented as mean ± SEM; ^a,b^ within a column, means without a common superscript differed (*p* < 0.05).

**Table A4:** Mean ± SEM effect of melatonin on methylation sequence of *DNMT3b*.

Group	CpG Position (%)							Sum (%)
	56	138	168:170	198	210	239	275	
C	36.1 ± 4.55 ^a^	15.4 ± 2.13	92.9 ± 8.81 ^a^	66.6 ± 5.37	37.2 ± 4.33	50.6 ± 4.22 ^a^	31.4 ± 3.25 ^a^	46.7 ± 5.79 ^a^
M	27.6 ± 3.01 ^b^	14.7 ± 1.64	68.4 ± 5.19 ^b^	51.4 ± 4.42	34.8 ± 3.47	38.7 ± 4.09 ^b^	20.6 ± 1.68 ^b^	36.0 ± 4.82 ^b^

The experiment was repeated three times; data presented as mean ± SEM; ^a,b^ within a column, means without a common superscript differed (*p* < 0.05).
